# A shape completion model for corrective osteotomy of distal radius malunion

**DOI:** 10.1007/s11548-025-03454-6

**Published:** 2025-06-17

**Authors:** Camiel J. Smees, Judith olde Heuvel, Stein van der Heide, Esmee D. van Uum, Anne J. H. Vochteloo, Gabriëlle J. M. Tuijthof

**Affiliations:** 1 Centre for Orthopaedic Surgery OCON, Hengelo, The Netherlands; 2 Department of Biomechanical Engineering, University of Twente, Enschede, The Netherlands

**Keywords:** Bone morphology, Corrective osteotomy, Radius malunion, Shape completion model, Statistical shape model, Surgical planning

## Abstract

**Purpose:**

When performing 3D planning for osteotomies in patients with distal radius malunion, the contralateral radius is commonly used as a template for reconstruction. However, in approximately 10% of the cases, the contralateral radius is not suitable for use. A shape completion model may provide an alternative by generating a healthy radius model based on the proximal part of the malunited bone. The aim of this study is to develop and clinically evaluate such a shape completion model.

**Method:**

A total of 100 segmented CT scans of healthy radii were used, with 80 scans used to train a statistical shape model (SSM). This SSM formed the base for a shape completion model capable of predicting the distal 12% based on the proximal 88%. Hyperparameters were optimized using 10 segmented 3D models, and the remaining 10 models were reserved for testing the performance of the shape completion model.

**Results:**

The shape completion model consistently produced clinically viable 3D reconstructions. The mean absolute errors between the predicted and corresponding reference models in the rotational errors were 2.6 ± 1.7° for radial inclination, 3.6 ± 2.2° for volar tilt, and 2.6 ± 2.8° for axial rotation. Translational errors were 0.7 ± 0.6 mm in dorsal shift, 0.8 ± 0.5 mm in radial shift, and 1.7 ± 1.1 mm in lengthening.

**Conclusion:**

This study successfully developed a shape completion model capable of reconstructing healthy 3D radius models based on the proximal bone. The observed errors indicate that the model is viable for use in 3D planning for patients lacking a healthy contralateral radius. However, routine use in patients with a healthy contralateral radius is not yet advised, as error margins exceed bilateral differences observed in healthy populations. The most clinically relevant error found in the model, length mismatch, can be easily corrected during 3D planning if the ipsilateral ulna remains intact.

## Introduction

Distal radius fractures are the most common fractures treated in emergency departments, with an incidence of 138 per 100,000 person-years [1]. Malunion is the most frequent complication of both conservative and surgical treatments for these fractures, occurring in up to 17% of cases [2, 3]. Symptomatic malunion is associated with pain, reduced mobility, and diminished grip strength, significantly affecting patients' quality of life [2, 4, 5]. Corrective osteotomy, a procedure in which the bone is cut and repositioned to restore its anatomical alignment, is an effective treatment for symptomatic malunion.

However, performing a corrective osteotomy can be challenging due to the multiplanar nature of the deformities [6, 7]. To address these challenges, the use of 3D technology for preoperative planning is rapidly gaining acceptance [8]. This approach facilitates precise visualization and planning, while 3D-printed patient-specific surgical guides for cutting and repositioning enhance intraoperative accuracy and improve surgical outcomes.

These 3D technologies typically rely on the contralateral healthy radius as a reference for the malunited radius. The process involves obtaining bilateral CT scans to segment the bones, mirroring the contralateral radius, and superimposing it onto the malunited radius. Differences between the two models are then used to create the surgical plan. A recent systematic review by Meesters et al. demonstrated that this 3D planning approach improves functional outcomes and reduces complication rates compared to conventional 2D methods [9].

Nevertheless, relying on the contralateral radius as a reference is not always feasible. Approximately 6% of distal radius fractures are bilateral [10]. Additionally, prior fractures or deformities in the contralateral radius further limit the applicability of the contralateral radius. At Centre for Orthopaedic Surgery OCON, we found that 7/107 (6.5%) of patients with symptomatic distal radius malunion lack a viable contralateral radius for reference. Even in cases where the contralateral radius is anatomically intact, bilateral anatomical differences may introduce errors into the surgical plan [11, 12]. A method enabling accurate 3D reconstruction without reliance on contralateral imaging could thus significantly improve surgical planning, reduce radiation exposure, and broaden the applicability of personalized surgical guides.

Shape completion models present a promising alternative for generating healthy bone models without requiring a contralateral reference [13, 14]. A shape completion model is a probabilistic model trained on datasets of healthy bone shapes, enabling the prediction of a new bone's shape based on partial input data. While shape completion models have been used in previous studies to reconstruct radii, these efforts primarily involved predicting large portions of the bone [13, 15]. For distal radius malunion, however, a model specifically tailored to predict the distal radius is required. As demonstrated by Mauler et al., predicting larger segments of the bone tends to result in greater local shape errors, as less information is available to predict the distal radius [13].

This study aims to develop a shape completion model capable of reconstructing a healthy distal radius using data from the proximal radius. Additionally, we seek to evaluate the model's performance and assess its clinical applicability.

## Methods

Following approval by the institutional review board, a retrospective search was performed to identify all patients at Centre for Orthopaedic Surgery OCON who received a bilateral CT scan for a radius malunion between 2018 and 2024. Patients were excluded if they met any of the following criteria: a non-healthy contralateral side, skeletal immaturity or a CT scan which does not span the entire radius. This selection process resulted in 100 patients.

The retrospectively collected CT scans had slice thicknesses of either 0.6 or 0.75 mm, and a B60s or Br59 CT kernel. The voltage was set at 120 kV, and the scan covered the area from proximal of the ulnar olecranon to distal of the radial styloid, ensuring the entire bone was imaged.

Per identified patient, the side without the malunion was semi-automatically segmented to create 3D bone models, using 3D medical imaging processing software Mimics (version 24.0, Materialise NV, Leuven, Belgium). These 3D bone models were then randomly assigned to one of three sets: training, validation and testing, with an 8:1:1 ratio. Demographic data, including age, sex, and the side of the malunion, were collected of all included patients.

### Development statistical shape model

**Fig. 1:**
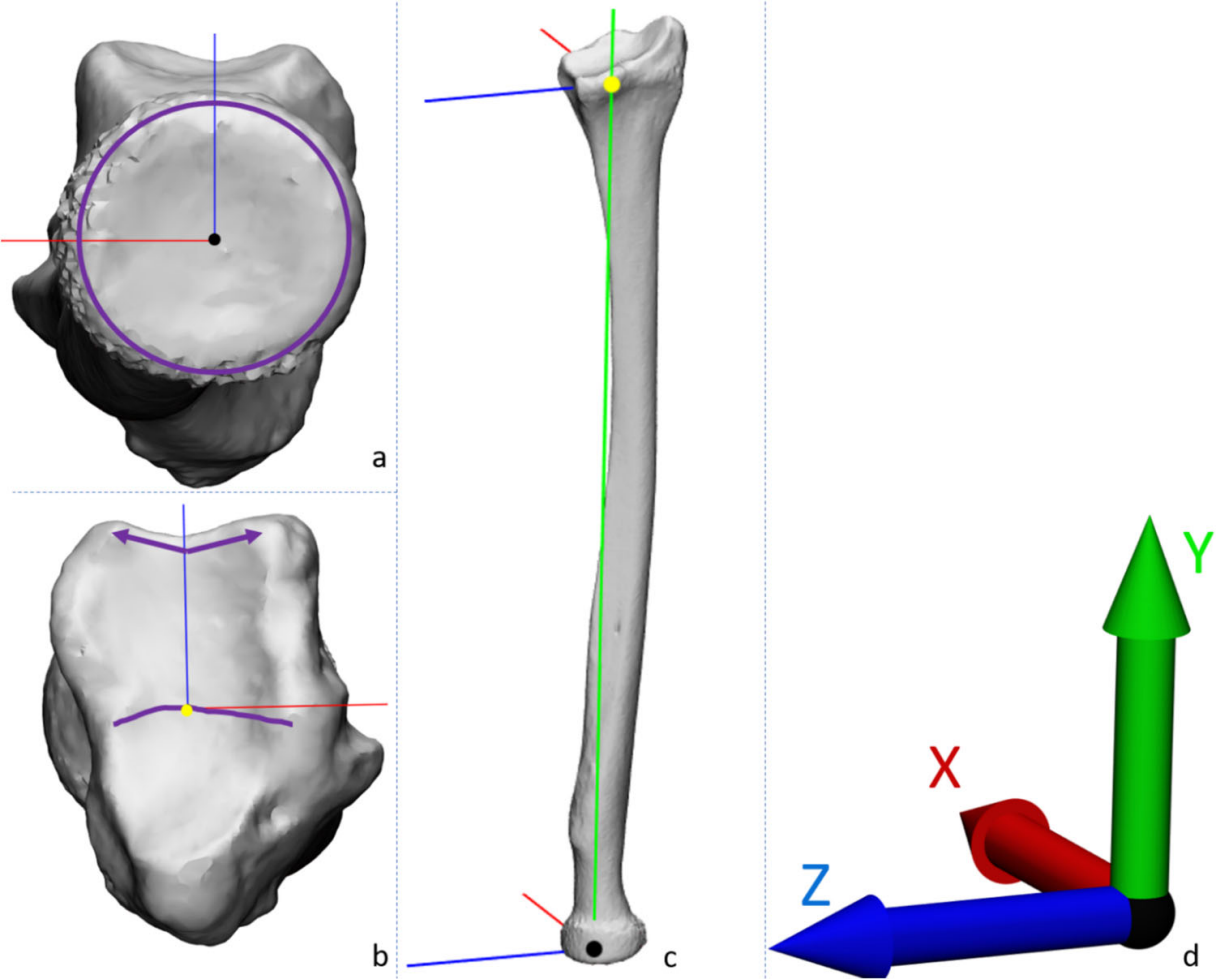
Definition of the coordinate system. **a** The origin of the coordinate system is located at the center of the radial head and is depicted in black. The edge of the radial head is displayed with a purple circle. **b** The distal end of the Y-axis is located halfway on the central ridge, highlighted with a purple line, of the distal radius. The Z-axis is perpendicular to the Y-axis and aligns with the middle of the sigmoid notch. Purple arrows indicate the distance between the middle of the sigmoid notch and its volar and dorsal ends. In yellow, a second origin of the coordinate system is represented which is used in the evaluation of the results. **c** An oblique view of the coordinate system. **d** The description of the colors of the coordinate system

The 3D bone models underwent pre-processing. Initially, all healthy left radii were mirrored to generate a dataset of 100 right radius 3D models. Subsequently, all 3D models were aligned with the coordinate system, as shown in Fig. 1. The coordinate system was defined such that the center of the radial head was positioned at the origin (Fig. 1a). The long axis of the radius running from this origin to the middle of the central ridge of the distal radius was aligned with the Y-axis, and the sigmoid notch was pointing in the Z-direction (Fig. 1b). The X-direction is perpendicular to both the Y and Z directions and points dorsally. For evaluation of the results, a second origin, represented by a yellow dot in the distal radius (Fig. 1b, c), was introduced to assess rotational and translational differences in the distal segment. The coordinate system is similar to the coordinate system described by Smees et al. [7], and an adaptation of the ISB recommended coordinate system [16].

The creation of the shape completion model began with the development of a statistical shape model (SSM) and was done following the work of Lüthi et al. [14]. The SSM was created using the open-source scripting library for statistical shape modeling and model-based image analysis Scalismo (University of Basel, Graphics and Vision Research Group). The final model is available upon reasonable request.

A downsampled radius 3D model, not included in the set of 100 3D models, was used as the reference for the SSM. Correspondence between each of the 80 3D models in the training set and the reference 3D model was established through nonrigid transformation. This was carried out in iterative steps using iterative closest point matching, where each subsequent step allowed for a larger possibility to nonrigidly deform.

After establishing correspondence between all 80 3D models and the reference, a generalized Procrustes analysis (GPA) was conducted to evaluate the shape variations within the dataset. The GPA provided the deformations between the reference 3D model and each of the 80 3D models. By averaging the deformation fields across all 80 3D models, the average deformation and consequently the mean shape of the dataset were determined. Additionally, principal component analysis was performed to identify the directions of the largest deformation among the 80 3D models, with the principal components ranked from largest to smallest deformation.

Using the mean shape as the reference 3D model, all steps were repeated—including correspondence establishment, GPA, and principal component analysis. The combination of the mean 3D model and the principal components together constituted the SSM.

### Development shape completion model

**Fig. 2:**
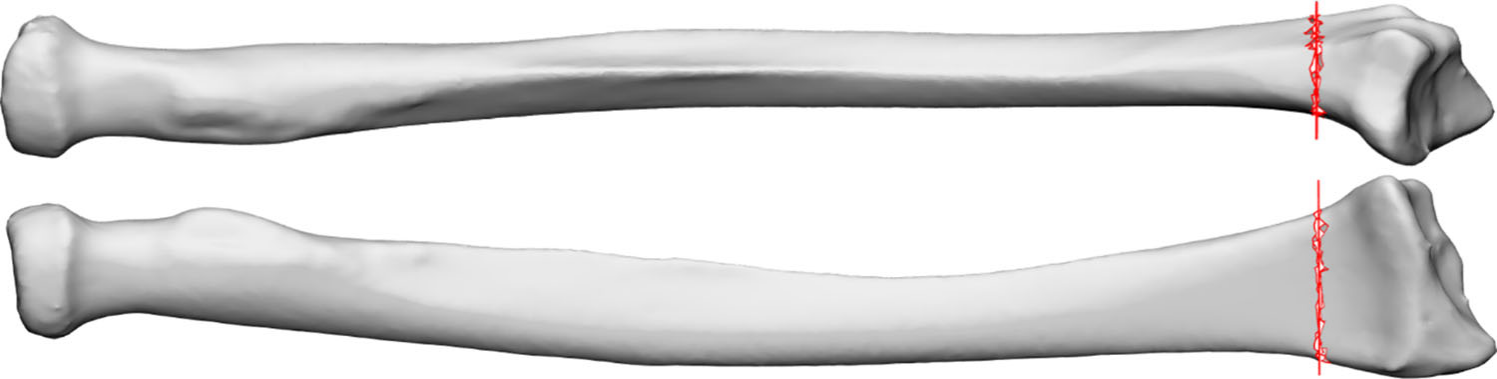
Cutoff location at 88% of the length of the radius, depicted in red. The radius is displayed from an ulnar (top) and a volar view (bottom)

Once the SSM was created, it was extended into a shape completion model using an isotropic Gaussian process model [14]. Specifically, Gaussian process regression was used, which utilizes the statistical correlations captured by the SSM to predict the missing portion of the bone from known partial shape data. This shape completion model was designed to predict the distal 12% of the radius, based on the proximal 88%. The 12% cutoff value was chosen to include most commonly seen metaphyseal extra-articular radius fracture locations [17]. The location of the cutoff height is depicted in Fig. 2.

A key step in creating the shape completion model was determining optimal hyperparameter values for the Gaussian process. These hyperparameters influence how well the model predicts missing shapes and must be manually tuned. They include noise, which represents the level of noise in the data; sigma, which controls the amount of correlation between neighboring points; and scale, which defines the size of the Gaussian kernel [14]. This hyperparameter tuning was performed systematically by evaluating noise levels of 5, 10, 15, and 20; sigma values of 50, 100, 150, 200, 250, and 300; and scale values of 5, 10, and 15. The optimal hyperparameter combination was identified as the one minimizing the mean prediction error.

To determine the values for the hyperparameters, the validation set was used. First, the distal 12% of each 3D bone model from the validation set was discarded. Next, each remaining proximal 88% of these 3D bone models was registered to the SSM’s mean shape to establish correspondence. Then, the shape completion model predicted the missing distal portion. The predicted complete 3D bone model including the 12% distal part was compared to its actual reference 3D model. The error between predicted and reference was quantified by calculating the mean difference as a Euclidean distance between both 3D models. This procedure was repeated for all ten 3D models of the validation set.

Once the optimal hyperparameter values were identified, the test set 3D models were used to evaluate the shape completion model. The test set was processed in the same manner as the validation set, but only with the optimal hyperparameter settings. The resulting predicted 3D models were then compared to the actual reference 3D models to determine the overall accuracy of the shape completion model.

### Evaluation

Evaluation of the shape completion model was conducted using measures of accuracy, compactness, generalization and specificity, as previously described by Audenaert et al. [18]. The accuracy of the shape completion model was determined as the mean distance between the shape completion model-generated 3D model and the reference 3D model for each point on the model’s surface. An accuracy smaller than the slice thickness of the CT scan, which was 0.6 mm, was deemed acceptable. The compactness of the shape completion model was evaluated by determining how many principal components were required to describe at least 95% of variation, as well as evaluation whether 100% of variation could be described. Generalization was assessed by incrementally determining the accuracy of the shape completion model as the number of 3D models in the training set increased. If the accuracy no longer improved, the shape completion model was considered population covering with that number of training 3D models. Specificity was evaluated by using the test set to generate 10 new 3D models and determining their accuracy and maximum Hausdorff distance. Additionally, heatmaps displaying the local differences between the shape completion model-generated 3D model and the test set actual reference 3D model were generated to evaluate local surface differences.

The results of the shape completion model were also expressed in clinically relevant measures: Three rotational (radial inclination; φx, volar tilt; φz, axial rotation; φy) and three translational (dorsal shift Δx, radial shift; Δz and lengthening; Δy) measurements were assessed. For each degree of freedom, the difference between the shape completion model-generated 3D model and the reference 3D model was determined. The origin of the coordinate system was aligned with the center of gravity of the distal segment of the reference 3D model (Fig. 1, yellow dot), and the orientation was aligned following the previously described coordinate system (Fig. 1). The distal segment was aligned with the shape completion model-generated 3D model through global registration and subsequent manual adjustment, similar to alignment in 3D surgical planning. The change in rotation and translation of the distal segment was used to determine the degrees of rotational and translational difference. Additionally, the 3D angle and 3D distance were calculated. These describe the directionally independent shortest rotation and translation between the shape completion model-generated 3D model and the test set 3D model.

To enable comparison with previous studies [13, 15], a second evaluation was conducted using a method similar to that employed in those studies. In this evaluation, the 3D model generated by the shape completion model was cut halfway along the Y-axis, which also served as the origin of the coordinate system for this analysis. The distal 50% of the predicted model was then aligned with the corresponding region of the reference 3D model using global registration. Rotational and translational differences were subsequently quantified based on the resulting transformation.

## Results

The study included 100 patients with a mean age of 46 years (SD 15). Of these patients 28 were male and 56 had a healthy right radius. The detailed demographics of the subsets are displayed in Table 1.

**Table 1:** Demographics of the entire dataset and the training, validation, and test subsets

	n	Age (years)	Male sex (n)	Healthy right side (n)
All	100	46 (SD 15)	28 (28%)	56 (56%)
Training	80	47 (SD 15)	23 (29%)	46 (58%)
Validation	10	39 (SD 17)	1 (10%)	4 (40%)
Test	10	46 (SD 10)	4 (40%)	6 (60%)

### Model parameters

**Fig. 3:**
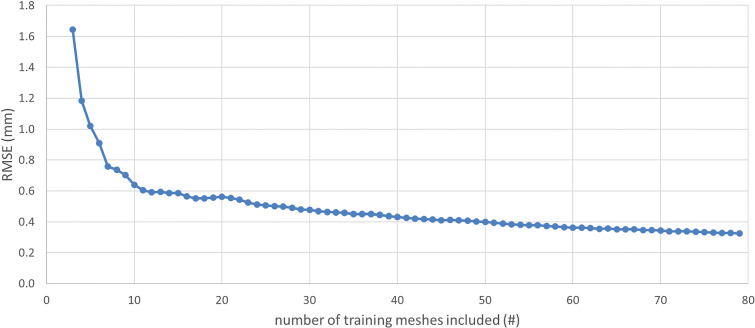
The generalization of the SSM is represented through the root-mean-square error (RMSE) as a function of the number of training meshes. Each dot represents the error between the reference model and the output of the SSM that was trained on the number of training meshes represented on the X-axis

The SSM was successfully developed. The generalization of the SSM is depicted in Fig. 3. The root-mean-square error (RMSE) decreased at a progressively slower rate as more training meshes were included. At 80 3D models included, the RMSE was 0.32 mm. The difference in RMSE between models 79 and 80 was 0.0032 mm.

During hyperparameter optimization of the shape completion model, the values that resulted in the best accuracy were a noise level of 20, a sigma of 100 and a scale of 5. With these parameter settings, the shape completion model achieved an average accuracy of 0.33 ± 0.06 mm on the validation set.

**Fig. 4:**
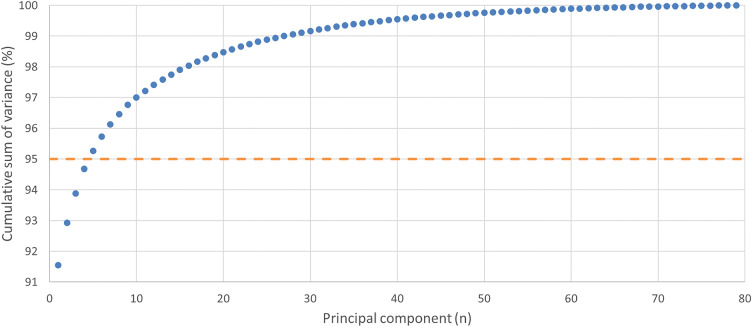
The compactness of the shape completion model, represented as the cumulative percentage of variance per number of principal components. The dotted line represents 95% of the variance

Using these hyperparameter settings, the shape completion model’s compactness, as illustrated in Fig. 4, showed that the first five principal components were sufficient to explain 95% of the variance in the dataset. Furthermore, the inclusion of 79 principal components accounted for 100% of the variance.

**Fig. 5:**
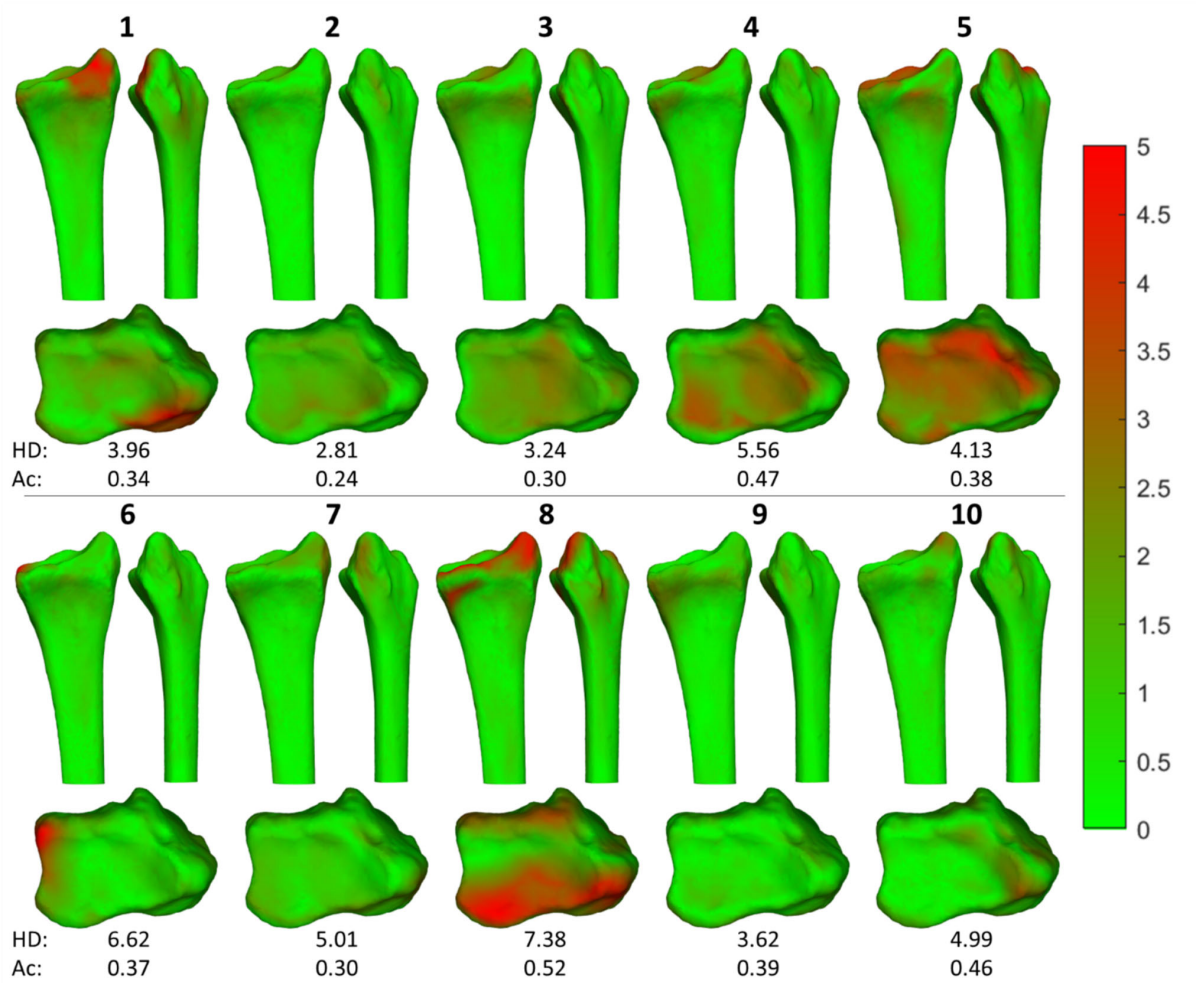
Differences between the shape completion model-generated 3D models and the corresponding reference 3D models in the test set. Per 3D model, a heatmap is presented in which the color represents the amount of distance between the shape completion model-generated 3D model and the reference 3D model at that location. Additionally, the maximum Hausdorff distance and the mean accuracy per 3D model are given. All values are depicted in mm. HD: maximum Hausdorff distance; Ac: Accuracy

The performance of the shape completion model is evaluated in Fig. 5. For each model in the test set, a heatmap was displayed, showing deviations in volar, radial and axial directions. Additionally, both the maximum Hausdorff distance and the mean accuracy were reported. Model 8 demonstrates the largest differences in the heatmap and had the highest maximum Hausdorff distance (7.38 mm). Generally, differences were observed in length and the distal volar surface.

### Clinical results

Differences of all individual rotational measurements were less than 10˚ between the shape completion model-generated 3D models and the corresponding test set reference 3D models (Table 2). Translational differences were predominantly observed in the lengthening direction (Δz) (Table 2). The largest rotational and translational differences were observed in model 8, consistent with the findings from the heatmaps (Fig. 5).

**Table 2:** Rotational and translational differences between the shape completion model-generated 3D model and the reference 3D model from the test set. Cases are displayed in order of increasing 3D angle difference. Mean and SD are based on absolute values

	Rotational differences (°)				Translational differences (mm)			
	3D angle	RI, φx	VT, φz	AR, φy	3D dist	DS, Δx	RS, Δz	Len, Δy
Case 5	2.9	− 1.9	− 1.8	1.0	3.1	1.6	− 0.3	− 2.7
Case 2	3.1	2.9	− 1.2	0.1	1.8	0.1	− 0.2	1.8
Case 3	3.3	1.1	1.0	3.0	2.2	0.0	− 0.4	2.2
Case 10	3.8	− 1.7	3.4	− 0.2	1.3	0.9	− 0.9	0.1
Case 7	4.5	− 2.4	− 3.7	0.5	1.0	− 0.6	0.6	0.5
Case 9	4.8	1.7	− 4.3	− 1.4	1.9	1.3	− 0.7	1.1
Case 6	7.4	6.5	− 1.7	3.1	1.1	− 0.3	− 0.7	− 0.8
Case 1	7.9	− 1.3	− 5.0	− 6.0	2.3	1.4	− 1.5	− 1.2
Case 4	10.3	5.3	− 8.7	1.1	3.4	− 0.2	0.4	3.4
Case 8	10.4	1.4	4.7	− 9.2	4.0	− 0.2	1.8	− 3.6
Mean	5.8	2.6	3.6	2.6	2.2	0.7	0.8	1.7
SD	2.8	1.7	2.2	2.8	1.0	0.6	0.5	1.1

*RI* Radial inclination; *VT* Volar tilt; *AR* Axial rotation; *3D dist* 3D distance; *DS* Dorsal shift; *RS* Radial shift; *Len* Lengthening; *SD* Standard deviation

The results of our shape completion model performance in which half of the shape completion model-generated 3D model was aligned with the reference 3D model can be found in Appendix Table 3.

## Discussion

This study aimed to develop a shape completion model to predict a healthy bone model, based on the unaffected segment of the ipsilateral radius. An SSM was created with an acceptable accuracy, which was the basis of the shape completion model. The shape completion model successfully predicted 3D radius shapes using the proximal bone segment (Fig. 5). The most clinically relevant residual error in the generated 3D models was a mismatch in length (Table 2).

The performance of the current shape completion model was compared to several shape completion models of the radius described in literature. Mauler et al. [13] described a model that generated the distal half of the radius based on the proximal half of the radius and the ulna. Oura et al. [15] described a model that generates 40% of the radius based on the proximal 60%. As previously described in the methods section, these studies used a larger bone segment in the evaluation of their model. The current shape completion model was evaluated using the same method as described in these studies. The results can be found in Appendix Table 3. In this comparison, the current shape completion model performed worse than Oura et al. with regards to axial rotation and lengtening, and worse than Mauler et al. with regards to lengthening, but outperformed both studies with regards to radial inclination, volar tilt, dorsal shift and radial shift. The maximum Hausdorff distance performed slightly worse than Mauler et al. and the accuracy of the model performed better than both previous studies.

While the results of the current model in comparison with previous models appears feasible, we believe that obtaining results in this way does not reflect clinical applicability of the shape completion model for distal radius malunion. By registering the distal 50% of the shape completion model-generated 3D model to the reference 3D model, a large residual error in the distal radius remains when the entire segment is registered. This is shown in Appendix Fig. 6. In contrast, when registering the distal 12% of the shape completion model-generated 3D model to the reference 3D model, the overlap in the articular surface is better, with less residual error in the registration. Therefore, the longer the segment is that is registered to the reference 3D model, the larger the residual error in the distal articular surface is. This occurs because larger segments constrain the alignment more strongly, reducing the degrees of freedom during registration. While this may improve the global alignment of the segment, it can mask local inaccuracies in clinically important regions such as the articular surface. As the reported outcomes are based on the transformation matrix obtained from this registration, a larger residual error means that the measured accuracy reflects alignment of the whole segment rather than local accuracy at the joint surface. As a result, models that predict longer segments may appear more accurate in global metrics, despite introducing greater error where it matters most.

To avoid this issue, the results presented in Table 2 were based on the registration of only the distal 12% of the radius. In our view, this provides a more clinically meaningful evaluation of the model’s accuracy, as it reflects the region relevant for surgical correction of distal radius malunion. Thus, we argue that models predicting longer segments are less suitable for localized applications, such as addressing distal radius malunion, as their reported error margins likely underestimate local morphological errors. This highlights the challenge of directly comparing outcomes between studies and underscores the importance of tailoring models to specific clinical problems, rather than relying on general models for local applications.

A comparison of the model’s performance to natural bilateral asymmetry of radii in a healthy population reveals mixed results [11]. Differences in radial inclination and volar tilt between the shape completion model-generated 3D model and the reference 3D model were larger compared to the left–right differences observed in a healthy population (2.6 ± 1.7° vs -0.6 ± 1.35° for radial inclination and 3.6 ± 2.2° vs 0.13 ± 1.00° for volar tilt), while the spread of axial rotation was smaller (2.6 ± 2.8° vs. 0.53 ± 5.00°). In terms of translational errors, dorsal shift was larger (0.7 ± 0.6 mm vs. -0.01 ± 0.64 mm), while radial shift and length errors were smaller (0.8 ± 0.5 mm vs -0.81 ± 1.22 mm and 1.7 ± 1.1 mm vs 2.63 ± 2.03 mm, respectively). These findings suggest that the model may not yet be suitable for patients with a healthy contralateral radius, as bilateral differences are smaller than the differences of the shape completion model in most directions. Nevertheless, our model’s errors remain below previously reported population-wide variation (radial inclination 3.0°, palmar tilt 4.1°) [20], suggesting that individualized predictions may still offer an advantage over general mean-based radius models, especially in patients lacking a healthy contralateral radius. However, this potential benefit should be tested in future studies by comparing anatomical accuracy between shape completion predictions and general mean models using ground-truth radius anatomy as reference.

The most clinically relevant error in the current model was lengthening, with seven out of ten predictions exceeding a 1 mm error (Table 2, Lengthening). There was no observable pattern describing which 3D models were generated longer or shorter compared to the reference. Nonetheless, in cases where the ulna is unaffected, the length of the radius can be corrected based on the length of the ulna [11, 19]. Since correction based on ulnar length is routinely performed in clinical practice during preoperative planning, this compensatory approach mitigates the impact of length discrepancies and enhances the model’s clinical applicability [19].

When evaluating the performance of the shape completion model, the results indicate high accuracy, with a mean accuracy of 0.33 ± 0.06 mm on the validation set 3D models. This is smaller than the slice thickness of 0.6 mm of the CT scans and therefore within an acceptable margin. The compactness of the SSM is high, as only 5 principal components are required to describe 95% of the variance. Since no comparative studies specifically for the radius were available, comparisons were made to other single-bone statistical shape models of long bones. Although differences in anatomical complexity and the number of included patients likely influence compactness outcomes, our model demonstrates favorable compactness in comparison. For instance, Fugit et al. [21] required 10 principal components for the femur and 18 for the tibia, while Audenaert et al. [18] required 20 for the femur and 21 for the tibia. Additionally, in our study 79 principal components were sufficient to describe 100% of the variance. The generalization showed a decrease in RMSE as more 3D models were included. Since the RMSE continued to decrease with the inclusion of the final 3D model, the dataset is not yet considered population covering, and additional 3D models should be incorporated. However, the decrease in RMSE is approaching zero, suggesting that only a small number of additional 3D models are required, with minimal improvement in RMSE expected.

To better understand the errors in the current shape completion model, an attempt was made to identify principal components that solely represented shape variations in the distal radius. It was hypothesized that determining the settings for these principal components would be challenging when relying solely on the proximal radius, potentially introducing uncertainty into the output of the shape completion model. The first 10 principal components were visually inspected to assess the type of shape variation each represented and to determine whether any principal components exclusively represented variation in the distal radius. None of the first ten principal components were found to meet this criterion.

A strength of this study was the use of a large dataset with proper validation and testing, in contrast to the leave-one-out approaches frequently reported in the literature [13, 15]. Furthermore, the model was specifically developed for distal radius malunion cases, enhancing its clinical relevance and applicability. The use of open-source software and the availability of the model upon request further promote transparency and encourage future research.

Despite these strengths, there are limitations to address. The margin of error of the shape completion model is larger than the bilateral differences in a healthy population, making the model unsuitable for patients with a healthy contralateral radius. If the model’s margin of error can be reduced below the bilateral differences, scanning of the contralateral radius is no longer needed, thereby reducing radiation exposure. Adding more models to the training set may be able to sufficiently lower the margin of error of the shape completion model, although the generalization (Fig. 3) indicates little expectable improvement of the model. An alternative way to reduce the margin of error may be incorporating orientation-independent distal shape data from non-fractured parts of the distal radius, as described by Sepasian et al. [22]. Including such data is expected to improve the predictive power of the model. Moreover, while the shape completion model is tested on healthy 3D radius models, further testing on 3D models from malunion patients is necessary to evaluate its performance in clinical practice. Nevertheless, as we tried to mimic the situation of a malunion as close as possible, we expect to find little deviations in this additional evaluation compare to the current study.

In conclusion, this study successfully developed an SSM-based shape completion model capable of predicting radius shapes using the proximal segment of the bone. The model demonstrated clinically acceptable margins of error in most cases, despite limitations in the accuracy of length prediction. These findings highlight the model’s potential as a tool for distal radius shape reconstruction, particularly in patients lacking a healthy contralateral reference. However, current error margins exceed bilateral differences seen in a healthy population, indicating that the model is not yet suitable as a replacement for contralateral-based registration. Although challenges remain, particularly in length prediction, future refinements—such as incorporating additional shape data and orientation-independent features—might enhance predictive accuracy and expand its clinical applicability. With further validation and optimization, this model has the potential to serve as a radiation-sparing alternative to contralateral registration, offering benefits to a broader patient population in the treatment of distal radius malunion.

## Figures and Tables

**Fig. 6:**
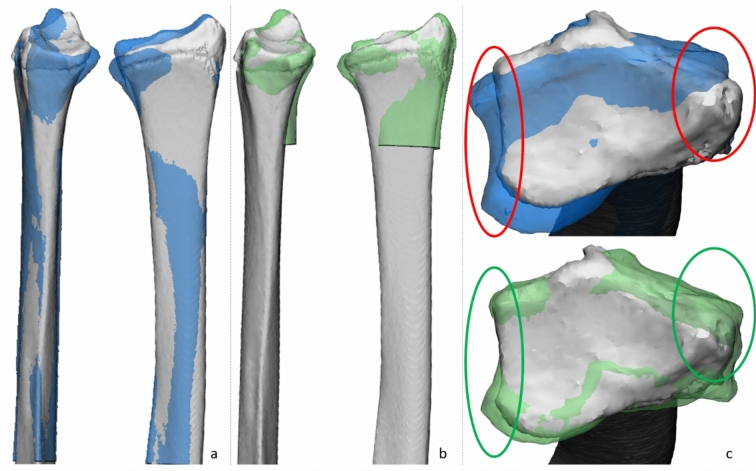
The registration of the shape completion model-generated 3D model to the reference model. **a** The shape completion model-generated 3D model (blue) is cut at 50% and registered to the reference model (white). **b** The shape completion model-generated 3D model (green) is cut at 88% and registered to the reference model (white). **c** The residual error in the distal radius of both methods is highlighted. In blue, a larger discrepancy between the shape completion model-generated 3D model and the reference 3D model is observed. In green, a smaller discrepancy between the shape completion model-generated 3D model and the reference 3D model is observed. Red and green outlines indicate the regions where the most notable differences in residual error are seen

## Data Availability

The code and data will be available upon reasonable request.

## Appendix

See Table 3 and Fig. 6.

**Table 3:** Rotational and translational differences between the shape completion modelgenerated 3D model and the reference 3D model from the test set, in which 50% of the model-generated 3D model was used in the registration. Mean and SD are based on absolute values. Results are displayed in comparison to previous studies of Mauler et al. [13] and Outra et al. [15]

	Rotational differences (˚)			Translational differences (mm)			Performance measures (mm)	
	RI, φx	VT, φz	AR, φy	DS, Δx	RS, Δz	Len, Δy	HD	Ac
Current								
Mean	0.2	0.2	1.7	0.1	0.1	1.6	4.7	0.4
SD	0.2	0.2	2.3	0.1	0.1	1.0	1.4	0.1
Mauler et al. [13]								
Mean	0.5	0.6	2.9	0.6	0.5	1.0	3.3	0.7
SD	0.4	0.5	2.2	0.5	0.4	0.8	1.2	0.1
Oura et al. [15]								
Mean	1.3	1.5	1.4	0.9	0.9	1.1	-	0.8
SD	1.1	1.2	1.3	0.8	0.7	0.9	-	0.3

*RI* Radial inclination; *VT* Volar tilt; *AR* Axial rotation; *DS* Dorsal shift; *RS* Radial shift; *Len* Lengthening; *HD* Maximum Hausdorff distance; *Ac* Accuracy; *SD* Standard deviation
